# Establishing a Universal RT-PCR Testing System Is Essential for the Safe Performance of Scheduled Surgeries During the COVID-19 Pandemic in Japan: A Retrospective Study

**DOI:** 10.7759/cureus.25974

**Published:** 2022-06-15

**Authors:** Tsuyoshi Nakai, Seizoh Nakata, Kiyonori Asao

**Affiliations:** 1 Orthopedics, Itami City Hospital, Itami, JPN; 2 Administration, Itami City Hospital, Itami, JPN; 3 Clinical Laboratory, Itami City Hospital, Itami, JPN

**Keywords:** elective orthopaedic surgery, covid-19 pandemic, covid-19, scheduled surgeries, rt-pcr (real time - reverse transcription polymerase chain reaction)

## Abstract

Background

Elective orthopedic surgery, as well as the procedures of many surgical departments, have been severely curtailed by coronavirus disease 2019. Here, we aimed to analyze how all surgeons could safely perform essential procedures during the coronavirus disease 2019 pandemic.

Methods

A retrospective review of elective surgeries performed between May 15, 2020, and February 28, 2022, was conducted. A screening questionnaire was used, and reverse transcription-polymerase chain reaction testing was assessed in all admitted surgical department patients. Their positivity rate and the positivity rate in our fever outpatient clinic were analyzed.

Results

Of 6099 patients who tested for severe acute respiratory syndrome coronavirus-2 during the study period, eight (0.13%) tested positive. The positive results were seen in four patients undergoing orthopedic surgery, two undergoing respiratory surgery, one undergoing breast surgery, and one undergoing plastic surgery. The number of patients who visited the outpatient clinic for fever was 15,639, including 1640 positive cases (positive rate of 10.5%). The positive rate of preoperative reverse transcription-polymerase chain reaction testing for scheduled surgery was consistently low and did not coincide with the peak of the wave of infection, while the positivity rate of outpatients with fever demonstrated a wave consistent with the national infection situation. All 6091 patients, excluding the eight positive patients, underwent surgery; all patients who underwent surgery were discharged from the hospital without developing coronavirus disease 2019 symptoms.

Conclusions

Our findings suggest that the establishment of a universal reverse transcription-polymerase chain reaction testing system is essential for the safe performance of scheduled surgeries during the coronavirus disease 2019 pandemic.

## Introduction

The rapid spread of coronavirus disease 2019 (COVID-19) led to it being declared a pandemic [1]. Furthermore, the repeated mutations of the severe acute respiratory syndrome coronavirus-2 (SARS-CoV-2) led to different waves of infections such as the Delta and Omicron. Japan was affected by five waves of infection and is currently in the middle of the sixth wave of COVID-19.

As the virus continues to mutate and further waves of infection are anticipated, countermeasures based on sufficient verification are necessary for orthopedic surgery to be performed safely. The number of orthopedic surgeries in our department has remained almost the same before and during the COVID-19 pandemic: 1466 in 2019, 1464 in 2020, and 1474 in 2021. This means that orthopedic surgery was necessary even during the COVID-19 pandemic; thus, surgeons are required to take sufficient countermeasures against COVID-19 and establish a system to safely perform surgery. Our hospital comprises 11 surgical departments, including orthopedics, digestive surgery, respiratory surgery, breast surgery, neurosurgery, obstetrics and gynecology, urology, plastic surgery, ophthalmology, dermatology, and dental surgery. All surgical departments are required to have a system in place to safely perform scheduled surgeries during the COVID-19 pandemic, not just orthopedics. Although there is little evidence regarding universal testing using reverse transcription-polymerase chain reaction (RT-PCR) for elective surgery in Japan, there are several reports from other countries [2-4].

The purpose of the present study was to report the percentage of positive RT-PCR tests for SARS-CoV-2 detected upon admission for elective surgery during the COVID-19 pandemic. We aimed to determine how all surgeons could safely perform essential procedures during the COVID-19 pandemic.

## Materials and methods

This retrospective observational study was conducted at a secondary care hospital in a city with a population of approximately 200,000. It was conducted in accordance with the principles laid down in the Declaration of Helsinki and following institutional review board approval (Ethical Review reference number 1645). In this study, a comprehensive agreement for academic use of the information such as type of treatments, treatment progress, or any other data acquired during their treatments was obtained from the patients by the hospital at the time of their hospitalization, and no identifiable information of the participants is included in the manuscript. In total, 6099 consecutive patients undergoing elective surgery between May 15 and February 28, 2022, were included in the study. Information regarding travel, occupation, contact, and cluster (TOCC) risk factors were documented for each patient. Screening for COVID-19 symptoms (fever, cough, dyspnea, myalgia or fatigue, headache, sneezing, sputum production, pharyngalgia, gastrointestinal discomfort, diarrhea, loss of appetite, and loss of taste or smell) was conducted, and the results were documented. If patients presented with any positive TOCC risk factors and/or exhibited symptoms, they were referred to a COVID-19 outpatient clinic. Thus, none of the included patients exhibited TOCC risk factors or COVID-19 symptoms.

RT-PCR testing was performed for all patients prior to scheduled surgery using samples taken from the nasopharynx 3-5 days prior to admission. The RT-PCR assays were performed using LightMix Modular SARS and Wuhan CoV E-gene/ Wuhan CoV N-gene (Roche Ltd, Basel, Switzerland). We chose this kit because its sensitivity and specificity are 100%, and we can expect a stable supply.

Due to the widespread of the COVID-19 in Japan, we believe that travel history is not a contributing factor and have removed travel history from the screening questionnaire after October 21, 2020 [3].

The hospital maintained a no visitor policy throughout the study period. At the fever outpatient clinic, COVID-19 antigen and RT-PCR tests were performed; if either test was positive, the patient was considered to be infected with COVID-19. In the fever outpatient clinic, the COVID-19 antigen assay kit used the Elecsys SARS-CoV-2 Antigen (Roche Ltd, Basel, Switzerland), while the COVID-19 PCR assay kit used the Film Arrey Respiratory Panel 2.1 (bioMérieux Japan Ltd, Tokyo, Japan) and Xpert Xpress SARS-Cov-2 Cepheid (Beckman Coulter Inc, California, United States) for diagnosis.

The positivity rates for scheduled surgical procedures and in the fever outpatient clinic were analyzed, and statistical analyses were performed using Excel statistics software (Microsoft Office 10; Microsoft, Seattle, USA); data are represented as percentages.

## Results

As shown in Table 1, among 6099 patients tested for SARS-CoV-2 during the study period, eight (0.13%) were positive: four orthopedic cases, two cases of respiratory surgery, one case of breast surgery, and one case of plastic surgery; none exhibited COVID-19 symptoms. The positivity rate of preoperative RT-PCR testing for scheduled surgery was consistently low and did not coincide with the peak of national infection.

**Table 1:** RT-PCR preoperative checks The RT-PCR-positive patients included four cases of orthopedic surgery, two cases of respiratory surgery, one case of breast surgery, and one case of plastic surgery. RT-PCR - reverse transcription-polymerase chain reaction

Year	Month	Number of patients	RT-PCR testing positive cases
2020	5	109	0
	6	242	0
	7	287	0
	8	270	0
	9	267	1
	10	278	0
	11	298	2
	12	278	0
2021	1	258	0
	2	304	0
	3	382	0
	4	285	0
	5	291	0
	6	289	0
	7	250	0
	8	313	2
	9	279	0
	10	279	0
	11	350	0
	12	256	0
2022	1	289	0
	2	245	3
Total		6099	8
Rate		0.13%	

**Figure 1:**
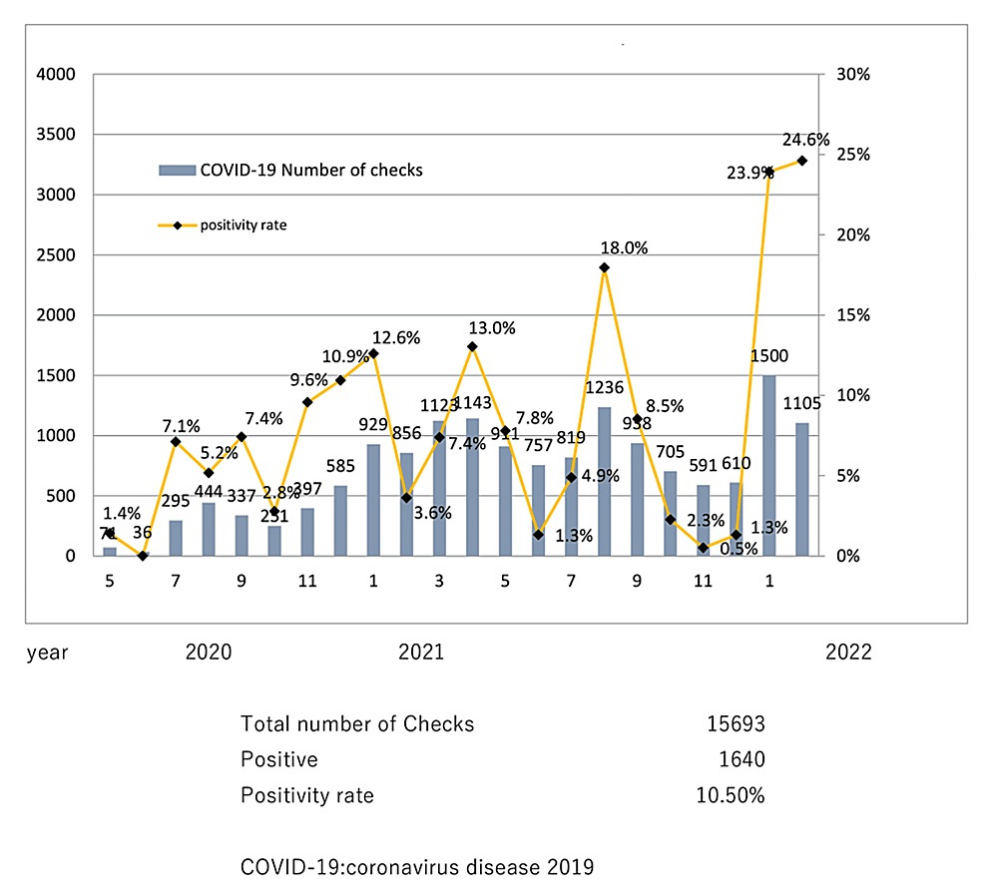
COVID-19 number of checks and positivity rate

In total, 6091 patients (the eight patients with positive results were excluded) underwent surgery; all patients who underwent surgery were discharged from the hospital without developing COVID-19 symptoms. Conversely, the number of patients who visited the outpatient clinic for fever was 15,639, including 1640 positive cases (positivity rate of 10.5%). Figure 1 shows the positivity rate of outpatients with fever, demonstrating a wave consistent with the national state of infection.

## Discussion

Our study revealed a COVID-19 incidence rate of 0.13% among the 6099 patients tested for SARS-CoV-2 during the study period. Among patients for scheduled surgery, eight tested positive between May 15 and February 28, 2022, using routine nasopharyngeal RT-PCR testing. These eight patients exhibited no COVID-19 symptoms.

It is possible that some patients who underwent surgery may have false-negative COVID-19 results. Therefore, the policy was to perform RT-PCR testing even after surgery if COVID-19 symptoms appeared.

The most important finding was that the positivity rate of preoperative RT-PCR testing for scheduled surgery was consistently low and did not coincide with the peak of national infection. The low positive rate may be due to the fact that patients who were scheduled to undergo surgery were supported to pay attention to hand hygiene, wear masks, and maintain social distance, and the patients also followed these guidelines. RT-PCR testing should be performed regardless of the peak of infection since it is possible that preoperative RT-PCR shows false-negative COVID-19 results.

Gruskay et al. reported that according to tests performed using preoperative nasopharyngeal swabs for COVID-19, 12.1% of patients were positive [5]. Contrastingly, we observed a low positivity rate in preoperative nasopharyngeal swab testing for COVID-19. However, this may be due to the relatively large number of patients tested in our cohort compared with that in their study.

San Miguel County, Colorado, reported that SARS CoV-2 IgG antibody tests were performed county-wide in March and April 2020. Twenty-nine of 5455 tested individuals (0.53%) were IgG positive, and 79 (1.45%) were borderline [6]. Our results demonstrated a low positivity rate for COVID-19, which may be due to vaccinations being promoted and the use of RT-PCR testing for COVID-19 diagnosis in our study, compared with the San Miguel study.

The American College of Surgeons recommends that elective surgery be considered essential. Maintaining access to surgery is an essential part of quality patient care, whether the surgery is needed to cure a medical condition, address infirmity, extend life, or contribute to patient well-being [7-9]. In our study, the 6091 patients who underwent elective surgery showed good clinical progress without any COVID-19 symptoms; hence, our goal was to analyze how all surgeons could perform essential procedures in a safe manner during the COVID-19 pandemic.

As the American College of Surgeons recommends that "decisions to adjust surgical services up or down should occur at a local level driven by hospital leaders including surgeons and in consultation with state government leaders; the decisions should be based on local case incidence, ongoing testing of staff and patients, aggressive use of appropriate personal protective equipment (PPE), and physical distancing practices", we advocate that elective surgery be performed after universal preoperative RT-PCR testing during the COVID-19 pandemic [7-9].

Our study has one notable limitation: it was conducted at a secondary emergency hospital located in a city with a population of approximately 200,000, thereby increasing the probability of selection bias. However, we can confidently assert that universal testing should be conducted.

## Conclusions

This study underscores the need for isolation of COVID-19 patients and their close contacts in the hospital. Thus, the identification of infected individuals is vital to prevent the spread of illness to other hospital personnel and patients. In conclusion, our findings suggest that the establishment of a universal reverse transcription-polymerase chain reaction testing system is essential for the safe performance of scheduled surgeries during the COVID-19 pandemic.
